# Vegetation response to exceptional global warmth during Oceanic Anoxic Event 2

**DOI:** 10.1038/s41467-018-06319-6

**Published:** 2018-09-20

**Authors:** Ulrich Heimhofer, Nina Wucherpfennig, Thierry Adatte, Stefan Schouten, Elke Schneebeli-Hermann, Silvia Gardin, Gerta Keller, Sarah Kentsch, Ariane Kujau

**Affiliations:** 1 Institute for Geology, Leibniz Universität Hannover, 30167 Hannover, Germany; 2 Institute of Geology and Palaeontology, Université de Lausanne, 1015 Lausanne, Switzerland; 3 Royal Netherlands Institute for Sea Research (NIOZ), Department of Marine Microbiology and Biogeochemistry, Utrecht University, 1790 AB Den Burg, Texel, The Netherlands; 4 Department of Earth Sciences, Utrecht University, 3584 CS Utrecht, The Netherlands; 5 Paleontological Institute and Museum, Universität Zürich, 8006 Zürich, Switzerland; 6 Centre de recherche sur la Paléobiodiversité et les Paléoenvironnements, Université Pierre et Marie Curie Paris 06, 75252 Paris, France; 7 Department of Geosciences, Princeton University, Princeton, 08544 NJ USA; 8 GFZ German Research Centre for Geosciences, Telegrafenberg, 14473 Potsdam Germany

## Abstract

The Cenomanian–Turonian Oceanic Anoxic Event (OAE2; ~94.5 million years ago) represents an episode of global-scale marine anoxia and biotic turnover, which corresponds to one of the warmest time intervals in the Phanerozoic. Despite its global significance, information on continental ecosystem response to this greenhouse episode is lacking. Here we present a terrestrial palynological record combined with marine-derived temperature data (TEX_86_) across an expanded OAE2 section from the Southern Provençal Basin, France. Despite high TEX_86_-derived temperature estimates reaching up to 38 °C, the continental hinterland did support a diverse vegetation, adapted to persist under elevated temperatures. A transient phase of climatic instability and cooling during OAE2 known as Plenus Cold Event (PCE) is marked by the proliferation of open, savanna-type vegetation rich in angiosperms at the expanse of conifer-dominated forest ecosystems. A rise in early representatives of Normapolles-type pollen during the PCE marks the initial radiation of this important angiosperm group.

## Abstract (web summary)

The Cretaceous Oceanic Anoxic Event 2 represents one of the warmest episodes in the last 250 million years. Here, the authors present spore-pollen data and temperature estimates (TEX_86_) across an expanded stratigraphic section illustrating the dynamic response of vegetation during this exceptionally warm interval.

## Introduction

Past time intervals of exceptional climatic warmth, typically associated with elevated *p*CO_2_, had profound impacts on floral compositions and biogeographic patterns of continental vegetation^1–3^. Within the overall greenhouse climate characterizing the Mesozoic, the Late Cretaceous Oceanic Anoxic Event (OAE) 2, which spans the Cenomanian–Turonian boundary [94.1 million years ago (Ma)], marks the onset of an extreme phase in ocean temperatures known as the “Cretaceous thermal maximum”^4–6^. This phase is characterized by one of the highest (>35 °C) proxy data-derived sea-surface temperature (SST) estimates of the last 150 Myrs, which are recorded by both planktonic foraminifera δ^18^O and archaeal membrane lipid-based TEX_86_ data^6^. A significant rise in low- and mid-latitude open ocean SSTs (2–4 °C) and mid-latitude shelf-sea temperatures (4–5 °C) accompanied the onset of OAE2, resulting in the Late Cenomanian–Turonian hothouse^5,7–9^. Besides the exceptional thermal conditions, the OAE2 (lasting 700–800 kyrs^10,11^) is associated with widespread formation of organic-rich deep-water deposits^12,13^, a major positive carbon isotope excursion (CIE) in carbonate and organic carbon reflecting massive burial of ^13^C-depleted carbon^14,15^, and major biotic turnover in marine ecosystems^16,17^.

Despite the outstanding position of OAE2 as one of the most remarkable events of the Mesozoic^18^, the responses of terrestrial ecosystems and continental flora to the changes of the global climate system remain largely unexplored. To date, just a few isolated plant macrofossil discoveries have been reported from the organic-rich Bonarelli Level, which is the sedimentary expression of the OAE2 in Marche–Umbria, Italy^19^. Microfloral evidence is essentially lacking due to the overwhelming predominance of amorphous kerogen in most marine OAE2 black shales, diluting any continent-derived spore-pollen signal. However, based on a shift in the δ^13^C signature of leaf-wax *n*-alkanes, a change from C3 to C4-dominated low-latitude vegetation triggered by a *p*CO_2_ drop has been proposed^20^ for the early phase of OAE2. According to theoretical considerations^21^, the exceptional warmth that prevailed during Late Cenomanian–Turonian times may have exceeded the heat tolerance of continental ecosystems, which potentially resulted in widespread vegetation dieback.

**Fig. 1:**
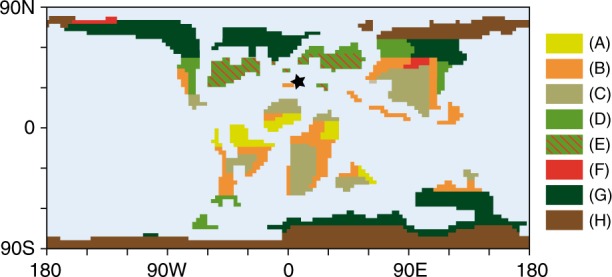
Palaeogeographic map illustrating Cenomanian–Turonian biome distribution. Palaeo-map is modified after ref. ^48^ and includes the extent of the Normapolles palynofloral province^29^. Color code representing (**A**) tropical moist, open canopy mixed forest with shrub understory; (**B**) savanna-type dry low understory with sparse trees; (**C**) deciduous dry/warm shrubland; (**D**) mid-latitude evergreen closed canopy conifer forest; (**E**) Normapolles province; (**F**) evergreen wet/cool shrubland; (**G**) high-latitude moist, open canopy forest with shrub understory; (**H**) boreal closed canopy conifer forest. Asterisk marks approximate location of the Southern Provençal Basin

Here we present a high resolution and taxonomically differentiated spore-pollen record across a stratigraphic interval correlating to the OAE2. The record is from an expanded marine section (Cassis) from the Southern Provençal Basin (SPB) of S’ France (Fig. 1) and has been analyzed for biostratigraphy, palynology, TEX_86_ and stable carbon isotopic composition of carbonates and organic materials. Stratigraphic assignment of the section is based on existing ammonite data^22^ combined with new information from planktonic foraminifera, calcareous nannoplankton, and carbon isotopes of bulk carbonate, organic matter and plant wax-derived long-chain *n*-alkanes.

## Results

### Stratigraphy

**Fig. 2:**
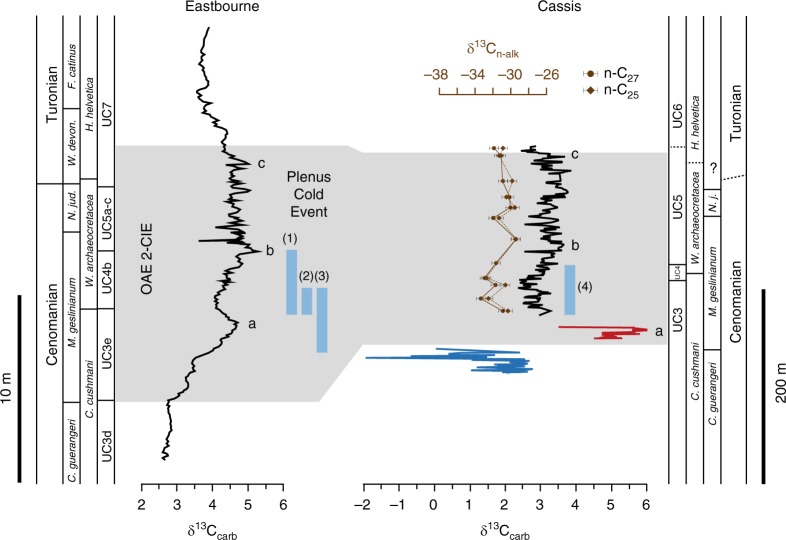
Bio- and chemostratigraphic correlation. Carbon isotope trend from Cassis, S’ France is compared with the European reference section at Eastbourne, Sussex, UK^23,24^. Calcareous nannofossil zonation of Eastbourne after ref. ^56^. Planktic foraminiferal biozonation of Eastbourne after ref. ^69^. Lowercase letters correspond to principal carbon-isotope peaks identified in the Eastbourne record^24^. Stratigraphic positions of the Plenus Cold Event (PCE) according to (1)^53^, (2)^70^, (3)^13^ with (4) representing supposed PCE position at Cassis. Color-code of the Cassis carbon-isotope record corresponds to lithostratigraphic formations^22,28^ with blue = Grès de l’Anse Sainte Magdeleine Fm., orange = Calcaires du Corton Fm., black = Marnes de l’Anse de l’Arène Fm. Carbon-isotope stratigraphic trend of plant-derived leaf-wax *n*-alkanes (*n*-C_25_; *n*-C_27_) is restricted to the Marnes de l’Anse de l’Arène Fm. δ^13^C values of individual *n*-alkanes are the means of duplicate runs (*σ* = ±0.5‰) expressed versus VPDB. Gray area represents the stratigraphic interval covered by the OAE2 carbon isotope excursion^24,54^. Note the expanded thickness of the OAE2 in the Cassis record

The ~235 m thick Cassis section represents a heterolithic succession with its lowermost part being composed of clays and marls overlain by (in part) turbiditic sandstones (0.0‒19.7 m). An exposure gap in the outcrop separates the siliclastic facies from a conspicuous limestone slope deposit characterized by intense slumping and disintegration (33.4‒46.1 m). Above another exposure gap, the main part of the section is continuously accessible (55.8‒236.0 m) and consists of homogenous marls with intercalated bundles of nodular limestone together representing basinal facies. Existing ammonite data combined with new biostratigraphic results constrain this section to the Upper Cenomanian–Lower Turonian^22^ (Supplementary Note 1). This age assignment is further corroborated and refined by carbon isotope data, which show a characteristic positive CIE revealing an initial high-amplitude positive peak (a) followed by a trough interval, which gives way to a plateau with two consecutive peaks (b, c) (Fig. 2). The characteristic pattern of the CIE is mimicked in the δ^13^C_org_ signature of bulk organic matter (Supplementary Fig. 1) and corroborated by the isotopic composition of long-chain *n*-alkanes derived from plant leaf-wax. Backed by high-resolution biostratigraphic data, the CIE evolution can be closely correlated with the reference carbon isotope record from Eastbourne, UK^23,24^ (Fig. 2). The significantly increased δ^13^C_carb_ values of the CIE peak (a) at Cassis (6.0‰) compared to Eastbourne (4.8‰) may reflect a shift in the dominant carbonate source in the SPB during this particular interval. In fact, the Calcaires du Corton Fm. corresponds to a calcareous unit composed of upper slope deposits, which can be traced toward the shoalwater carbonate platform bordering the SPB to the north where similarly high δ^13^C_carb_ signatures are observed in OAE2-equivalent platform limestones^25^. Carbonate carbon isotope signatures > 6.0‰ for the OAE2 CIE are also described from the epeiric Iberian seaway^26^ and may reflect variations in the aragonite content of the periplatform ooze exported from the adjacent platform^27^. The correlation thus reveals very good stratigraphic coverage of the uppermost Cenomanian–Turonian at Cassis with an expanded ~200 m thick OAE2 interval (Fig. 2). High sedimentation rates accompanied by increased input of continental organic debris in a transtensive geotectonic setting^28^ may have prevented the accumulation of black shales enriched in marine-derived organic carbon in the SPB.

### Palynology

At Cassis, high numbers of inaperturate and bisaccate gymnosperm pollen (avg. = 48.2%) are essentially produced by Araucariaceae and Cupressaceae-type plants (*Araucariacites* spp.; *Inaperturopollenites* spp.) and Cheirolepidiaceae (*Classopollis* spp.) with subordinate contributions from Pinaceae (*Cerebropollenites* spp.) and Podocarpaceae (*Podocarpidites* spp.). Other gymnosperms, including cycads-ginkgophytes (*Cycadopites* spp.), gnetaleans (*Ephedripites* spp.), and seed ferns (*Alisporites* spp.) are quantitatively of minor importance (avg. = 2.5%). Spores produced by a diverse assemblage of ground ferns and fern allies occur in moderate quantities (avg. = 12.8%). The assemblage contains a variety of eudicotyledonous angiosperm pollen represented by various tricolporate and triporate types, the majority of which can be assigned to early forms of the Normapolles complex (predominantly *Atlantopollis* and *Complexiopollis* groups; avg. = 18.9%) (Supplementary Fig. 2).

**Fig. 3:**
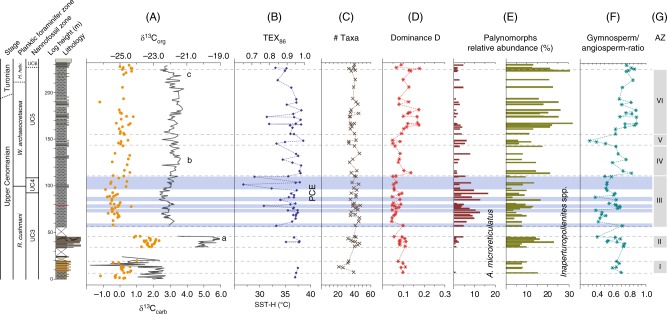
Terrestrial palynomorph and TEX_86_ data across OAE2. Stratigraphic trends in δ^13^C_carb_, δ^13^C_org_, TEX_86_-derived sea-surface temperatures (SSTs) and evolution of palynofloral diversity and composition across OAE2 at Cassis, S’ France. Biostratigraphy and lithological log of Cassis section to the left. (**A**) Carbon-isotope stratigraphy based on bulk rock carbonate and organic matter; (**B**) TEX_86_ and SST reconstruction based on TEX_86_^H^ calibration; (**C**) species richness; (**D**) dominance index; (**E**) stratigraphic distribution of selected pollen including the Normapolles-type angiosperm pollen *Atlantopollis reticulatus* and the conifer pollen *Inaperturopollenites* spp.; (**F**) ratio of gymnosperm versus angiosperm taxa; (**G**) assemblage zones (AZs) based on characteristic spore-pollen associations. PCE = Plenus Cold Event. Blue horizontal bands represent individual SST cooling episodes during the PCE

Based on stratigraphic variations in relative spore-pollen abundances, six characteristic assemblage zones (AZ I–VI) are differentiated from base to top (Fig. 3). A pre-CIE assemblage (AZ I, 6.5–18.5 m) shows the lowest number of individual taxa (avg. = 30.2) and a moderately high gymnosperm to angiosperm (G/A) ratio (avg. = 0.65) with the angiosperm component being dominated by non-Normapolles-type pollen. Among conifer pollen, relatively high abundances of *Cerebropollenites* spp. (avg. = 7.3%) are paralleled by low *Inaperturopollenites* spp. content (avg. = 9.5%). The overlying assemblage (AZ II, 34.7–45.2 m) corresponds to the first build-up phase (peak a) of the CIE and shows increased total taxa counts (avg. = 39.9). An increase in Normapolles-type angiosperm pollen results in slightly lower G/A-ratios (avg. = 0.62). Conifer-derived *Inaperturopollenites* spp. remain similar (avg. = 10.6) but show a marked increase toward the top of AZ II reaching up to 22.9%. Above, the trough-shaped CIE interval corresponds to an assemblage (AZ III, 57.1–110.8 m) characterized by stable numbers of taxa (avg. = 40.1) and a low dominance index with average D values of 0.06 indicating a high degree of ecological evenness of the floral association. Increased abundances of Normapolles-type pollen (e.g., *A. microreticulatus*) are paralleled by low conifer pollen contents (e.g., *Inaperturopollenites* spp.) resulting in comparatively low G/A-ratios (avg. = 0.55). The onset of peak (b) is again characterized by an assemblage rich in conifer-derived grains and low angiosperm pollen (AZ IV, 114.2–143.2 m) with increased G/A-ratios (avg. = 0.70) and high dominance index (avg. = 0.09). Another increase in Normapolles-type pollen marks the overlying assemblage (AZ V, 146.3–149.4 m) which shows the lowest G/A-ratios (avg. = 0.41) paralleled by low D values of 0.06. Above, the upper part of the CIE plateau up to peak (c) corresponds to the topmost assemblage (AZ VI, 156.7–225.3 m). Here, the strong dominance of a few abundant conifer-derived pollen (with *Inaperturopollenites* spp. reaching up to 31.8%; avg. = 20.4%) causes substantially higher G/A-ratios (avg. = 0.77) and higher dominance indices (avg. = 0.12). Calculated origination and extinction records show very low values and the range-through diversity comparatively high and stable values throughout the studied interval, which are illustrating the absence of major land plant extinction during OAE2. Increased values at the base of the origination record and at the top of the extinction record, as well as reduced values at the base and the top of the range-through diversity trend are interpreted as consequences of edge effects.

The overall rich and diverse palynological association obtained from Cassis is taxonomically distinctive for a position within the Late Cretaceous Normapolles phytogeographic province^29^. Stratigraphic trends in the distribution of the abovementioned groups and indices are considered to primarily reflect changes in the vegetation structure of the adjacent continental hinterland of the SPB.

### TEX_86_ temperature estimates

SST estimates based on TEX_86_ show continuously high temperatures >35 °C for the SPB during Late Cenomanian–Turonian times with maximum SSTs reaching 38 °C (Fig. 3). These warm SSTs conform with existing TEX_86_ studies from early Late Cretaceous mid- and low-latitude sites^5,6,8,9^ and highlight the exceptional temperature regime prevailing during deposition of OAE2. A gradual SST decline is indicated by decreasing TEX_86_ values characterizing the latest Cenomanian to earliest Turonian at Cassis. In the SPB, the overall pattern of exceptional high temperatures is punctuated by several transient drops towards significantly lower TEX_86_ values (<0.8), which translate into SSTs below 32 °C. These cooler SST estimates characterize a phase of climatic instability and occur within an interval stratigraphically placed within the upper UC3 and UC4 nannofossil zone, below and close to the *R. cushmani*-*W. archaeocretacea* zone boundary and corresponds to the trough-shaped segment of the CIE between peaks (a) and (b).

## Discussion

Critically high temperatures have been put forward as a mechanism for the suppression of terrestrial plant and animal life during the end-Permian extinction and basal Triassic events^30–32^. For these intervals, exceptional climatic warmth is considered a first order control for the well-documented turnover in continental vegetation^3,33^. During the Cretaceous thermal maximum, exceptional warmth and associated heat stress have also been suggested to significantly affect plant growth by inhibiting photosynthesis to a certain extent at daytime temperatures ranging between 35° and 42 °C^21^. Continental temperatures in this range are considered life-limiting to plants, resulting in serious thermal stress and potential die-off^34,35^. However, the rich and diverse flora reconstructed from palynological datasets across the OAE2 does not support the idea of widespread heat-induced die-off in continental vegetation—at least not in the hinterland of the studied SPB. Despite maximum TEX_86_-derived SSTs of up to 38 °C reconstructed for the SPB, the hinterland supported a rich and diverse flora, which is roughly similar in composition to the type of vegetation thriving in Late Cenomanian to Early Turonian times in other parts of the palaeo-European archipelago^36,37^. Mid-latitude terrestrial plant ecosystems were apparently well adapted to the exceptional warm conditions prevailing during the Cretaceous thermal maximum. The potential effects of temperature extremes might have been more aggravated in continental interior regions of the subtropics and close to the equator, where even higher mean annual surface temperatures are to be expected^38^.

Despite the absence of major plant extinction associated with OAE2, variations in the relative contribution of individual taxa and groups reflect compositional changes in vegetation. High contents of Araucariaceae and Cupressaceae-derived pollen indicate the presence of forest communities composed of large trees forming a dense emergent cover^39,40^. An arborescent habit of Cretaceous araucarioids is supported by abundant fossil wood finds from mid-latitudes^41^ and in line with the growth habit of modern Araucaria types^42^. Together with high pollen abundances produced by Pinaceae, Podocarpaceae and xerophytic Cheirolepidiceae, this type of assemblage (AZ II, IV, and VI) is interpreted to reflect mixed forest dominated by arborescent conifers with an only moderate angiosperm component, which grew in mesic habitats with moderate availability of moisture. Such conifer-dominated ecosystems prevailed during episodes of increased δ^13^C values including the first build-up (peak a, AZ II), the second build-up (peak b, AZ IV) and upper plateau phase (AZ VI) of the CIE. During the trough-shaped CIE segment and between peaks b and c, conifer forests were replaced by more diverse, angiosperm-rich vegetation with increased contents of Normapolles-derived pollen (AZ III and V). Late Cenomanian taxa assigned to *Atlantopollis* and *Complexiopollis* are the first representatives of the Normapolles complex^36,43^, an angiosperm group, which dominated mid-latitude northern hemisphere assemblages during the Late Cretaceous and Early Cenozoic. Based on in-situ findings in fossil flowers, pollen of the Normapolles-type is related to core Fagales^44^. The thick, often multi-layered exine and complex apertures indicate anemophily^45^ and are considered adaptions to open vegetation with widely spaced trees under more dry to seasonally dry climates^46^. During the late Cenomanian, Normapolles-producing angiosperms were probably small non-woody plants or shrubs forming part of a xerophytic savanna-type vegetation^47,48^—a view supported by the rarity of fossil dicot wood in Cenomanian mid-latitude strata from Europe^49^. Accordingly, the palynological record across OAE2 shows changing proportion of arborescent conifer forests (reflecting more mesic conditions) and more open, non-arborescent, angiosperm-rich vegetation.

**Fig. 4:**
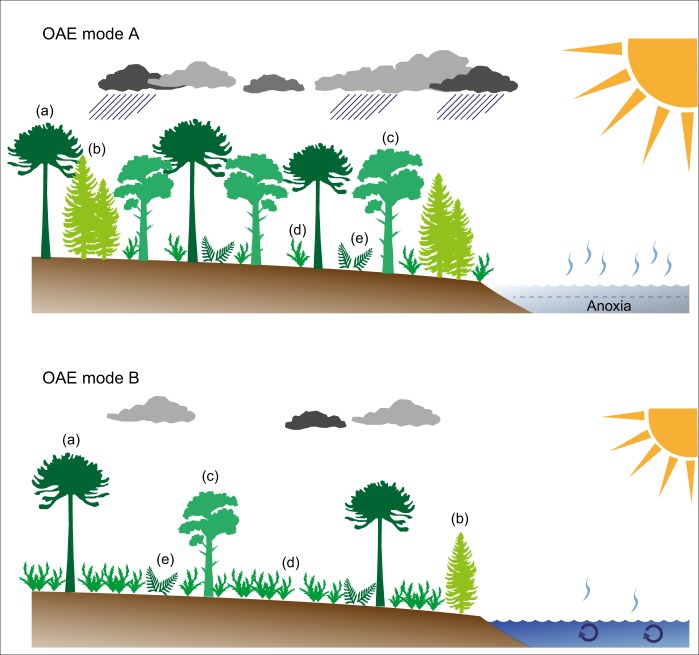
Tentative changes in mid-latitude vegetation patterns during OAE2. During OAE mode A (e.g. represented by AZ II, IV and VI), the prevailing climate was characterized by exceptional warmth and high moisture availability, giving way to conifer-dominated forests with moderate angiosperm contribution. During OAE mode B (AZ III and V), climatic conditions were cooler and less humid resulting in an open, savanna-type vegetation community with increased abundances of Normapolles-producing angiosperms. (**a**) Araucariaceae, (**b**) other conifers incl. Cheirolepidiaceae, (**c**) Cupressaceae, (**d**) angiosperms incl. Normapolles-producing forms, (**e**) ferns

Fluctuations in the overall climatic patterns during OAE2 have been reported earlier based on marine-derived proxy data. Exceptional climatic warmth associated with OAE2 is considered to have caused accelerated hydrological cycling, enhanced weathering and a generally more humid climate^9,50–52^. Phases of enhanced moisture availability may have fostered the spread of mesic conifer-dominated forest ecosystems during the onset and later phase of OAE2. Such warm and humid conditions were punctuated by a series of pronounced climatic coolings during the so-called Plenus Cold Event, PCE^53,54^. The PCE represents a phase of climatic instability and is characterized by several short-lasting SST drops and increases in the range of 2.5–11 °C^5–13^ and paralleled by the southward migration of boreal fauna^55^. Climatic instability was accompanied by a shift towards significantly drier conditions in northern hemisphere mid-latitudes^9^. Stratigraphically, the main pulse of the PCE is located above the first CIE build-up (peak a) at Eastbourne^54^, where it corresponds approximately to the boundary between the *R. cushmani* and *W. archaeocretacea* zones and UC3 to UC4 transition, respectively^56^. At Cassis, the PCE is expressed in a series of several transient drops of TEX_86_-derived SSTs below 32 °C with the strongest SST decline occurring within the upper part of the CIE trough interval (Fig. 3), similar to what has been observed elsewhere^5^. This part of the CIE with a shift towards lower δ^13^C values has been related to improved bottom-water oxygenation, enhanced organic carbon remineralization and significant fluctuations in atmospheric *p*CO_2_^13,54,57,58^. In continental mid-latitude settings such as Cassis, this phase of climatic upheaval is characterized by the proliferation of open, savanna-type vegetation rich in angiosperms at the expanse of conifer-dominated forest ecosystems (Fig. 4). Once established, the savanna-type biome was able to persist even during recurrent phases of exceptional warmth in the course of the PCE.

The onset of the Cretaceous thermal maximum including OAE2 took place against the background of a major turnover in global land plant vegetation, namely the rise of angiosperms towards ecological dominance. In mid-latitude settings of North America and Europe, this floral change is marked by the Late Cenomanian radiation of Normapolles-producing plants related to core Fagales^43,45^. In the Cassis record, early representatives of this group show a pronounced increase in the uppermost Cenomanian assemblage zone III, subsequent to their Mid- to Late Cenomanian origination^36^. Within the overall warm and humid greenhouse conditions characterizing OAE2, a transient shift towards relatively drier climates and punctuated cooling during the PCE may have fostered a first spread of Normapolles-type angiosperms, which henceforward became a very diverse and successful group dominating mid-latitude plant ecosystems throughout the Late Cretaceous to Early Cenozoic for more than ~50 Ma.

## Methods

### Stable isotope analyses of carbonate and organic carbon

Measurements of stable carbon and oxygen isotopes of sedimentary carbonates were carried out on powdered bulk rock material (~0.5 mg) on a total of 292 samples. Stable isotope analysis was conducted using a Thermo Fisher Scientific Gasbench II carbonate device connected to a Thermo Fisher Scientific Delta V Advantage IRMS, available at the Leibniz University Hannover, Germany. The gas bench uses viscous water-free (98 g/mol) orthophosphoric acid at 72 °C to release CO_2_ of the calcite from the sample material 1 h before the start of the measurement. Repeated analyses of certified carbonate standards (CO-1, NBS-18, NBS-19) show an external reproducibility ± 0.08‰ for δ^18^O and ± 0.06‰ for δ^13^C_carb_. Values are expressed in conventional delta notation relative to the Vienna-Pee Dee Formation belemnite (VPDB) international standard, in per mil (‰) Stable carbon isotope analyses of bulk organic carbon (δ^13^C_org_) were performed on 117 decarbonated samples. Powdered samples were treated twice with 6 M HCl for 12 h to remove any carbonate phases and rinsed subsequently with deionised H_2_O until neutrality was reached. Stable carbon isotope composition of bulk C_org_ was determined using an organic elemental analyser (Thermo Scientific Flash 2000) connected online to a Thermo Fisher Scientific Delta V Advantage IRMS, available at the Leibniz University Hannover, Germany. The analytical accuracy and reproducibility is checked by replicate analyses of international standards (NBS 22). Reproducibility was better than ±0.1‰ for δ^13^C_org_. Values are expressed in conventional delta notation relative to the Vienna-Pee Dee Formation belemnite (VPDB) international standard, in per mil (‰).

### Palynology

A total of 67 rock samples from the Cassis section were prepared for palynological analysis by the Geological Survey of North Rhine-Westphalia in Krefeld, Germany. Cleaned, crushed and weighed samples (20 to 50 g) were treated with 30% HCl and 38% HF for carbonate and silica removal, respectively. Residues were sieved over a 11-μm mesh and mounted on microscope slides, which were analyzed at ×200 and ×1000 magnification. All samples were productive and studied for their particle content (palynofacies) and sporomorph assemblage (spores and pollen), respectively. For the quantification of the spore-pollen assemblage, a minimum of 250 grains were determined per slide (avg. 272 grains) and one entire slide was scanned for rare elements. Five samples did not provide enough sporomorphs to reach the target count of 250 grains. Variations in the spore-pollen assemblage represent normalized frequencies and are reported in percentage [%] of the total assemblage. Thermally unaltered preservation of organic matter is indicated by the virtually unchanged coloring of the sporomorphs and shows a thermal alteration index (TAI) < 2^59^. Preservation of the individual pollen grains varies from moderate to good. Sporomorphs are assigned to floral groups according to their botanical affinity^60,61^.

### Biomarker analysis

For TEX_86_ analysis, a total of 69 powdered and freeze dried samples (5–10 g dry mass) were extracted with an accelerated solvent extractor, using a 9:1 (v/v) dichlormethane (DCM):MeOH solvent mixture, 3 times for 5 min. at a pressure of ca. 7.6 × 10^6^ Pa and a temperature of 100 °C. The obtained total extracts were rotary evaporated and separated over an activated Al_2_O_3_ column using 9:1 (v/v) hexane:DCM and 1:1 (v/v) DCM:MeOH solvent mixtures into an apolar and polar fraction, respectively. The polar fraction, containing the glycerol dialkyl glycerol tetraether lipids (GDGTs), was dried under a pure N_2_ flow, dissolved ultrasonically in a 99:1 (v/v) hexane:isopropanol mixture at a concentration of 2 mg/ml and filtered over an 0.45 μm mesh PTFE filter (Ø 4 mm) prior to HPLC/MS analysis. High-performance liquid chromatography/atmospheric pressure chemical ionization mass spectrometry (HPLC/APCI-MS) analyses was be performed on an Agilent 1100 series/Hewlett-Packard 1100 MSD series machine equipped with an auto-injector and HP Chemostation software following^62^.

GDGTs were quantified by integration of peak areas and the TetraEther indeX of tetraethers composed of 86 carbon atoms (TEX_86_) was determined^63^. In order to assess the influence of soil-derived GDGTs on TEX_86_ values, the BIT index^64^ was applied. Samples with BIT index > 0.3 were excluded from SST reconstruction^65^. Absolute SSTs were reconstructed for 64 samples using TEX_86_^H^ core top calibration equations^66^, which has a calibration error of 2.5 °C. The analytical error of TEX_86_^H^-based SST estimates was 0.07 °C based on duplicate analysis. We used the TEX_86_^H^ calibration rather than other calibrations^67^ in order to remain consistent with previous literature^6^ and as this has recently been shown to match independent temperature estimates in tropical regions during the Eocene hothouse, the warmest period in the Cenozoic^68^. Although the uncertainty of the calibration used is 2.5 °C, comparisons with other proxy datasets^6^ suggests that TEX_86_ sometimes can overestimate SSTs, or have a seasonal bias, e.g., toward summer temperatures. Nevertheless, even considering this, the here presented SST estimates of up to 38 °C do suggest hot tropical temperatures well above 30 °C. A level-by-level comparison of TEX_86_ values and G/A-ratios based on 42 samples supports a general link between SSTs and gross vegetation patterns (Supplementary Fig. 3).

For compound-specific carbon isotope measurements, apolar fractions were treated using an Ag-Silica column with hexane and subsequently ethyl acetate to gain a mostly *n*-alkane pure fraction, which was evaporated under N_2_ stream followed by dissolution in 25–50 µl hexane. For measuring the δ^13^C composition of individual *n*-alkanes, 1 ml was injected into the gas chromatograph isotope ratio mass spectrometer (Agilent 6890N GC coupled to a Thermo Delta V advantage IRMS), equipped with a fuse silica capillary column coated with CP-Sil5, with helium used as a carrier gas. The oven was programmed at a starting (injection) temperature of 70 °C, which rose to 130 °C at 20°/min and then 320° at 4°/min, at which it was maintained for 20 min. Values are expressed in conventional delta notation relative to the Vienna-Pee Dee Formation belemnite (VPDB) international standard, in per mil (‰). All biomarker analyses were carried out at the NIOZ (Royal Netherlands Institute of Sea Research, The Netherlands).

## Electronic supplementary material

Supplementary Information

## Data Availability

All data generated and/or analyzed in this study are included in this published article and its supplementary information file, and are also available from the corresponding author on reasonable request.
