# Supplementary material for: Vegetation response to exceptional global warmth during Oceanic Anoxic Event 2
Source: Nat Commun. 2018 Sep 20;9:3832. doi: 10.1038/s41467-018-06319-6 (PMC6148089; doi:10.1038/s41467-018-06319-6)
Supplement: Supplementary file 1 — Supplementary Information [file 41467_2018_6319_MOESM1_ESM.pdf]

Vegetation response to exceptional global warmth during Oceanic Anoxic Event 2

Heimhofer et al.

Supplementary Note 1

Stratigraphy

The ~235 m thick Cassis section is exposed along the coastline stretching from the town of Cassis towards the SE. The base of the succession is located 500 m SE of the Cassis marina at Pointe de Lombards (N 043°12'34''; E 005°32'23''). From here, the stratigraphic succession can be followed from the Anse Sainte Magdeleine cove past the Pointe de Corton peninsula towards the Anse de l'Arène cove and up the steep barren slopes below the cliffs of the Cap Canaille (N 043°12'20''; E 005°33'03''). Lithostratigraphy follows refs. 1 and 2. The studied section starts above an erosional unconformity (d1 in ref. 2), which separates the marls and claystones of the Marnes de Cassis Fm. from the overlying Grès de l'Anse Sainte Magdeleine Fm. (0.0—19.7 m), which resembles a heterolithic sequence of calcareous silt- and sandstones with few intercalated marly beds. Following a gap in outcrop due to the low erosional resistance of the overlying Marls de l'Anse Sainte Magdeleine Fm., the intensively slumped calcareous marls and limestones of the Calcaires du Corton Fm. (33.4—46.1 m) form a conspicuous lithological unit well exposed along the Pointe de Corton peninsula. Above another section gap, an extended succession of homogenous grey marls occurs, which is interrupted by several bundles (labelled a-f) of nodular limestone corresponding to the Marls de l'Anse de Arène Fm. (55.8—236.0 m). Bundles are composed of up to 20 individual limestone layers. A conspicuous yellowish cm-thick horizon in the lower part is interpreted as altered bentonite layer.

Based on ammonite findings, the succession can be assigned to the Upper Cenomanian (*Calycoceras guerangeri*, *Metoicoceras geslinianum*, *Neocardioceras judii* ammonite zones) and the Lower Turonian (*Mammites nodosoides* ammonite zone)[1]. Existing and novel planktonic foraminifera data from the Marnes de l'Anse de l'Arène Formation identify the *Rotalipora cushmani*, *Whiteinella archaeocretacea* and *Helvetoglobotruncana helvetica* biozones, thereby constraining this part of the section to the uppermost Cenomanian to lowermost Turonian. These results are further corroborated by calcareous nannoplankton data with the assemblage showing more close affinities with boreal assemblages then compared to Tethyan ones. Stratigraphically important nannofossil bioevents include the

last occurrences (LOs) of *Corollithion kennedyi*, *Lithraphidites acutum* and *Axopodorhabdus albianus* as well as the first occurrences (FOs) of *Cylindralithus biarcus* and *Quadrum intermedium*. *Helenea chiastia* and *Rhagodiscus asper* are present up to the top of the studied section. The nannofossil assemblage enables a refined stratigraphic subdivision and assignment to the UC3 to UC5b nannofossil zones[3].

Carbon isotopes derived from bulk carbonate vary between 0.0 and 2.7 ‰ (avg. of 1.7 ‰) within the more siliciclastic-rich deposits in the lowermost part (0.0—19.7 m), with few negative $\delta^{13}C$ peaks as low as -1.9 ‰ recorded in the sandstone-rich facies. The above-lying slumped limestone interval (33.4—46.1 m) shows a prominent positive shift with peak values in the upper part reaching 6.0 ‰. The marls of the Marnes de l'Anse de l'Arène Fm. are characterized by less positive values ranging between 2.4 and 3.9 ‰. Here, the lower part extending from 61.9 to 118.9 m (corresponding to the UC3, UC4 and lowermost UC5 nannofossil zones) shows a trough-shaped $\delta^{13}C$ trend with lowest values reaching 2.4 ‰. This is followed by an extended interval (119.0—224.2 m) characterized by more positive values (avg. of 3.4 ‰) forming a relatively stable plateau, only terminated at the very top (224.3—236.0 m) by a subtle decline to $\delta^{13}C$ values of ~2.7 ‰. The $\delta^{13}C$ pattern observed in bulk carbonate is mimicked in the carbon isotope composition of sedimentary bulk organic carbon as well as in the long-chain *n*-alkanes of probable leaf wax origin. Bulk rock sedimentary organic matters shows values around -25 ‰ in lowermost part (0.0—19.7 m), followed by distinctively less negative values (up to -22.7 ‰) in the slumped interval represented by the Calcaires de Corton Fm. (peak a). Above, the trough-shaped trend in the carbon isotope record is well expressed in the $\delta^{13}C_{org}$ record with values reaching as low as -25.8 ‰ at around the UC3 to UC4 transition. Similar to the carbonate $\delta^{13}C$ trend, the overlying deposits (119.0—224.2 m) are characterized by less negative $\delta^{13}C_{org}$ values fluctuating around -24.5 ‰. The *n*-alkanes show most negative values of -33.4 ‰ in the trough-shaped segment between peaks a and b (avg. of -32.1 ‰ for n-C_{27}). Above, the *n*-alkane signature is less negative and averages -30.8 ‰ for n-C_{27}. No long-chain *n*-alkane $\delta^{13}C$ data was obtained from the Calcaires de Corton Fm. and below lying strata due to low *n*-alkane concentrations.

Supplementary Figure 1

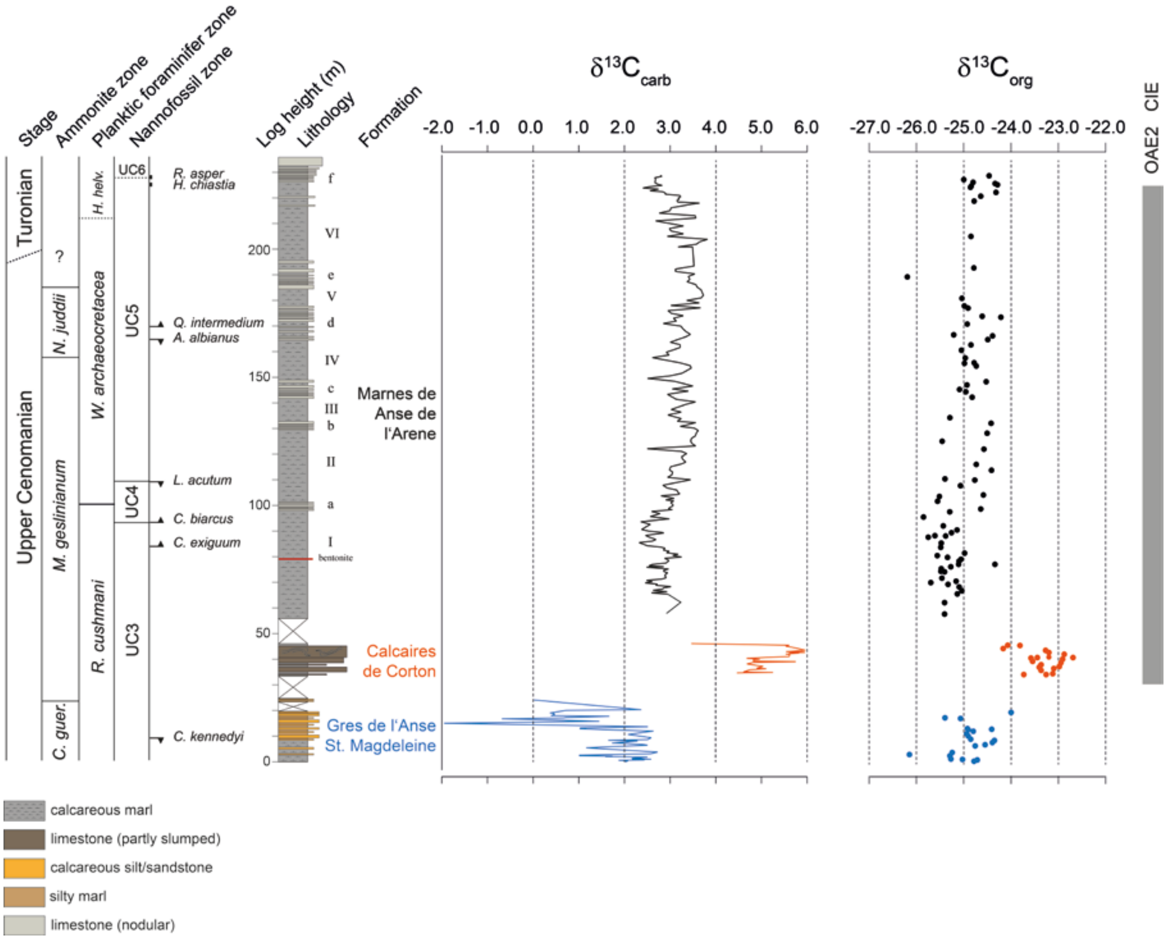

Supplementary Figure 1 – Lithological log, biostratigraphy and δ^{13}C chemostratigraphy of the Cassis section, SE France. Ammonite zonation according to [1]. *C. guer.* = *Calycoceras guerangeri*; *H. helv.* = *Helvetoglobotruncana helvetica*.

Supplementary Figure 2

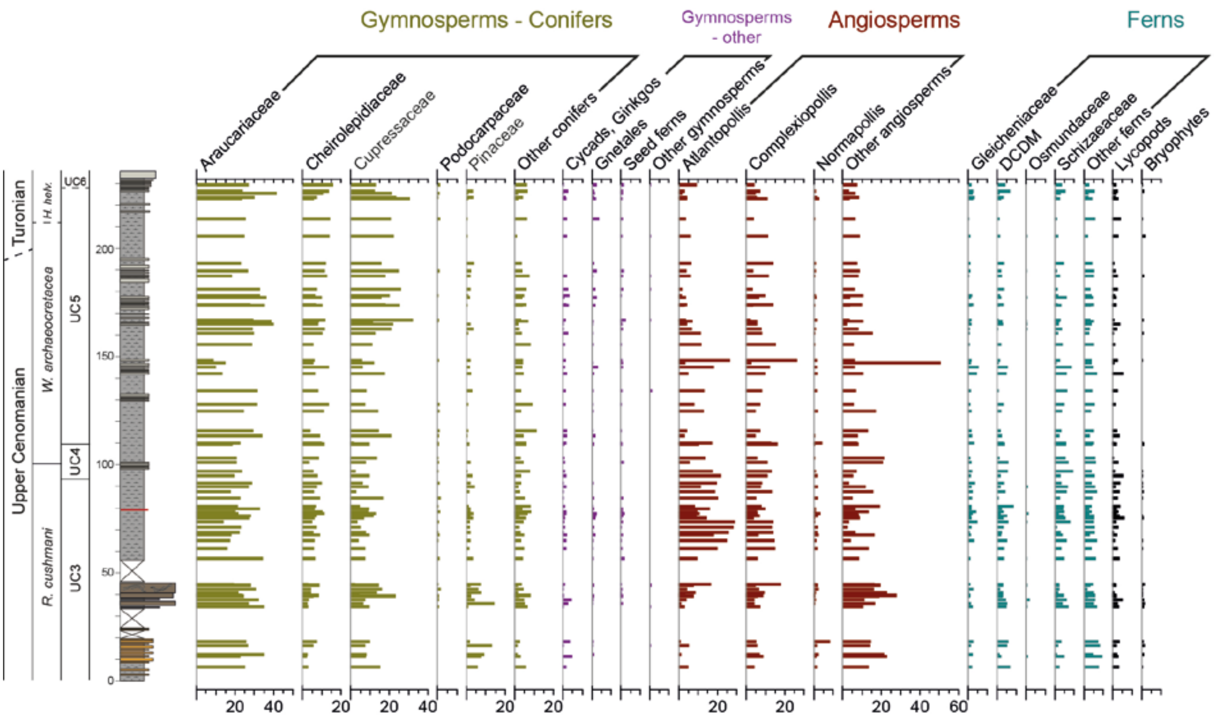

Supplementary Figure 2 – Lithological log, biostratigraphy and stratigraphic frequency distribution (relative abundance in %) of pollen and spores grouped according to botanical affinity following refs. 4 and 5.

Supplementary Figure 3

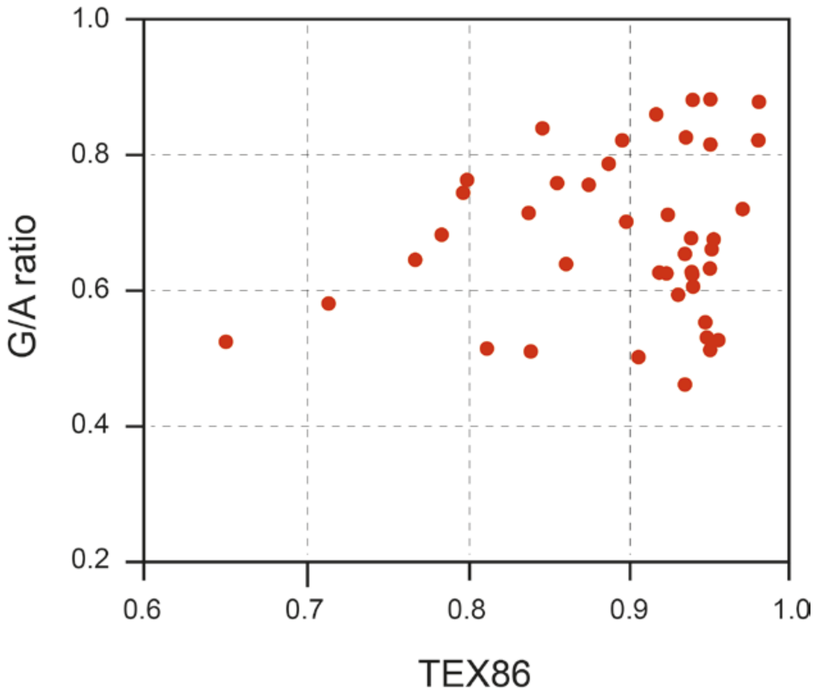

Supplementary Figure 3 – Cross-plot of TEX_{86} values and gymnosperm/angiosperm (G/A) ratios from 42 samples derived from the similar stratigraphic level.

Supplementary references

1. Jolet, P., Philip, J., Thomel, G., López, G., Tronchetti, G. Nouvelles données biostratigraphiques sur la limite Cénomanien–Turonien. La coupe de Cassis (Sud-Est de la France): Proposition d'un hypostratotype européen. *Geobios* **34,** 225–238 (2001).
2. Floquet, M., Gari, J., Hennuy, J., Léonide, P., Philip, J. Sédimentations gravitaires carbonatées et silicoclastiques dans un bassin en transtension, séries d'âge Cénomanien à Coniacien moyen du Bassin Sud-Provençal. *Field guide of the 10ème Congrès Francais de Sédimentologie,* 1–80 (2005).
3. Burnett, J.A. in *Calcareous Nannofossil Biostratigraphy* (ed Bown, P. R.) 132–199 (Chapman and Hall, London, 1998).
4. Abbink, O. A., Van Konijnenburg - Van Cittert, J. H. A., Visscher, H. A sporomorph ecogroup model for the northwest European Jurassic–lower Cretaceous: Concepts and framework. *Geol. Mijnbouw* **83,** 17–31 (2004).
5. Balme, B. E. Fossil in situ spores and pollen grains: An annotated catalogue. *Rev. Palaeobot. Palynol.* **87,** 81–323 (1995).
